# Norms for Zung’s Self-rating Anxiety Scale

**DOI:** 10.1186/s12888-019-2427-6

**Published:** 2020-02-28

**Authors:** Debra A. Dunstan, Ned Scott

**Affiliations:** School of Psychology University of New England, Armidale, NSW 2351 Australia

**Keywords:** Anxiety screening, Zung self-rating anxiety scale (SAS), Cut-off score

## Abstract

**Background:**

Zung’s Self-rating Anxiety Scale (SAS) is a norm-referenced scale which enjoys widespread use a screener for anxiety disorders. However, recent research (Dunstan DA and Scott N, Depress Res Treat 2018:9250972, 2018) has questioned whether the existing cut-off for identifying the presence of a disorder might be lower than ideal.

**Method:**

The current study explored this issue by examining sensitivity and specificity figures against diagnoses made on the basis of the Patient Health Questionnaire (PHQ) in clinical and community samples. The community sample consisted of 210 participants recruited to be representative of the Australian adult population. The clinical sample consisted of a further 141 adults receiving treatment from a mental health professional for some form of anxiety disorder.

**Results:**

Mathematical formulas, including Youden’s Index and the Receiver Operating Characteristics Curve, applied to positive PHQ diagnoses (presence of a disorder) from the clinical sample and negative PHQ diagnoses (absence of a disorder) from the community sample suggested that the ideal cut-off point lies between the current and original points recommended by Zung.

**Conclusions:**

Consideration of prevalence rates and of the potential costs of false negative and false positive diagnoses, suggests that, while the current cut-off of 36 might be appropriate in the context of clinical screening, the original raw score cut-off of 40 would be most appropriate when the SAS is used in research.

## Background

Along with depression, anxiety disorders are the most prevalent of mental health conditions [1, 2]. Formal diagnoses based on the Diagnostic and Statistical Manual of Mental Disorders (DSM [3];) require a clinical interview, but as this is a time-consuming and expensive process, clinicians and researchers employ a variety of psychometric tools to screen for these conditions. These screeners include both criterion-referenced measures, such as the Patient Health Questionnaire (PHQ [4];), and norm-referenced measures, such as the Depression Anxiety Stress Scale (DASS [5];), the Beck Anxiety Inventory (BAI [6];), and the State-Trait Anxiety Inventory (STAI [7];), which allow comparison of the individual’s results with a norm-refernced group of sufferers. In the latter case, scores that equal or exceed a specified cut-off point are considered to indicate the likely presence of the disorder concerned. While it is beyond the scope of this paper to critique the psychometric properties of such norm-referenced screeners or compare their advantages, disadvantages and limitations, users should be mindful of such features [8–13].

The paper focuses on Zung’s Self-rating Anxiety Scale (SAS [14];) a norm-referenced screener that, in conjunction with its sister scale, the Self-rating Depression Scale (SDS, [15]) has been shown to discriminate anxiety from mood disorders [16]. Although developed in 1971, the SAS continues to be extensively used in research, particularly in medical disciplines [17]. The SAS has good psychometric credentials [11, 14] and has been found to perform comparatively to contempory measures such as the anxiety subscale of the DASS in predicting anxiety disorder classifications based on the PHQ [16]. However, two problems have emerged in the literature regarding the Zung SAS cut-off score to indicate the presence of a disorder. These are: the use of an index score [14] and a change in Zung’s recommended cut-off point [14, 18, 19].

Zung developed a method of scoring both the SDS [15] and SAS [14] that involved conversion of a total scale raw score (with a potentail range of 20 to 80) to a index score with a potential range of 25 to 100. The index score is ‘derived by dividing the sum of the values (raw scores) obtained on the 20 items by the maximum possible score of 80, converted to a decimal and multiplied by 100’ [14] (p. 376). Within current research, Dunstan and Scott [17] have identified confusion between raw scores and the index scores with many reseachers failing to perform the raw-score-to-index-score conversions recommended by Zung. This has led to misclassification of participants in up to 45% of studies in which the SAS has been employed [17].

In 1980, Zung [18] reduced the cut-off point for clinical significance from that set in his seminal paper on the development of the SAS [14]. Recent research has suggested that the 1980-recommended cut-off point (a raw score of 36 or an index score of 45 [18];) is lower than ideal, and that the original 1971 cut-off (a raw score of 40; an index score of 50 [14];) produces better sensitivity and specificity figures [16]. Similar problems; that is, confusion between index and raw scores and suggestions that the currently recommended cut-off score may be too low, have also been identified for the SDS [16, 17, 20]. This current study explores this issue for the SAS. Specifically, is the raw score^1^ cut-off of 36 most recently recommended by Zung appropriate or should it too be increased?

To avoid furthering the confusion between raw and index scores, from this point on, only raw scores will be used in this paper. This includes scores taken from Zung’s research which have been converted back to their raw score form.

### Methods for setting cut-off scores

Zung [18] states that his recommended cut-off for the SAS was chosen with reference to the means and standard deviation of the clinical and normal adult population samples used. The precise criteria used here are not clear: the mean SAS score for the clinical population was 47.0 (*S.D.* = 9.5) and for the normal adult population 33.4 (*S.D.* = 7.8).

Aside from examining means and standard deviations of populations with and without the condition in question, other commonly used methods to determine clinical cut-off points include the Youden Index and the Receiver Operating Characteristics (ROC) curve [21]. The Youden Index method is designed to give equal weight to sensitivity and specificity: that is false positives and false negatives are treated as equally undesirable [22, 23]. Youden’s Index for a cut-off point equals the sum of the sensitivity (Se) and specificity (Sp: expressed as probabilities) minus one (Se + Sp – 1). The cut-off is set at the score which yields the highest Youden Index value. The ROC curve method graphs Se (on the y-axis) versus 1 – Sp (on the x-axis). The point (0,1) on the ROC curve represents a test which perfectly distinguishes between positive and negative diagnoses: that is specificity and sensitivity are both 100%. The closer the curve approaches this point, the better the test. Hence, another method is to set the cut-off as the value for which the ROC curve is closest to that point [24]. This method places more emphasis on achieving a balance between sensitivity and specificity values. However, a further alternative is to set the cut-off to correspond to the point where the curve intersects the line Se = Sp: thereby obtaining the best possible balance between sensitivity and specificity [21]. ROC curves also provide a measure of a test’s overall discriminatory ability: the greater the area under the curve, the stronger the test [24]. While the use of ROC curves has its origins in medical disease diagnosis, ROC curves have been successfully applied to explore optimal cut-off scores for psychological screeners (e.g., [20, 25, 26]).

### The current study

The study conducted by Dunstan et al. [16] employed a sample primarily composed of undergraduate psychology students at a regional Australian university, complimented by a small clinical sample. As such, its findings were not considered robust or representative enough to determine whether a change in the SAS cut-off score was appropriate. The current study sought to further explore the appropriate cut-off for the SAS by exploring sensitivity and specificity, as determined by PHQ classifications, amongst representative community and clinical samples of the Australian population.

## Method

### Participants

Two separate samples of participants, all aged 18 and over, were recruited from Qualtrics survey panels. The community sample was recruited to be representative of the broad Australian adult community and consisted of 210 participants (108 men and 102 women) with a mean age of 45.59 years (*SD* = 17.43, range = 18–82). Participants who were receiving treatment from a mental health professional for either a depressive or anxiety disorder were expressly excluded from this sample. The clinical sample consisted of a further 141 adults (49 men and 91 women with a mean age of 42.55 [*SD* = 15.95, range = 18–79]) receiving treatment from a mental health professional for some form of anxiety disorder.

Further details of the demographic features of the participants are shown in Table 1. Individuals with a diagnosis of mental illness involving psychotic features, or who had experienced a major loss in the last six months, were excluded from the study as were those who could not read/understand English.

**Table 1:** *Sample Demographics*

	Clinical Sample (*n* = 141)	Community Sample (*n* = 210)
Place of birth	%	%
Australia	82	65
Europe	11	13
New Zealand	1	6
Middle East	3	1
Rest of Asia	1	10
Rest of Africa	1	2
USA/Canada	1	*
Not stated	1	2
Highest education level		
Year 10 or less	13	19
Year 12	16	17
TAFE/Trade qualification	42	37
Undergraduate degree	17	21
Postgraduate degree	12	6
Employment Status		
Full-time	21	23
Part-time	23	18
Casual	7	10
Unemployed	32	27
Retired	17	23
Current Occupation		
Manager	9	9
Professional	11	9
Technician/Tradesman	3	4
Clerical/Admin	8	8
Machinery/Transport	–	1
Labourer	3	5
Community/Personal Serv	4	2
Sales	5	7
Other	9	6
None	49	50
Household Income		
Less than 1 k	51	41
1 – 3 k	37	34
3 k plus	5	11
Not stated	7	15

### Procedure

The survey was distributed to Qualtrics panel members who first answered a series of questions to confirm their eligibility. Those eligible were then asked to complete a short 10- min online survey consisting of demographic and biological information plus the two scales detailed below. Participants were free to opt out of the study at any time.

### Measures

#### Zung self-rating anxiety scale (SAS)

The Zung SAS is a self-report scale whose 20 items cover a variety of anxiety symptoms, both psychological (e.g, *“I feel afraid for no reason at all”* and *“I feel like I’m falling apart and going to pieces”*) and somatic (e.g., *“My arms and legs shake and tremble”* and *“I feel my heart beating fast.”*) in nature. Responses are given on a 4-point scale which range from 1 (*none, or a little of the time*) to 4 (*most, or all of the time*). Participants are instructed to base their answers on their experiences over the last week. Items include both negative and positive (e.g., *“I fall asleep easily and get a good night’s sleep.”*) experiences, with the latter being reverse scored. Raw scale scores for the SAS range from 20 to 80. The SAS has satisfactory psychometric properties. These include: internal consistency (Cronbach’s alpha = .82) [11]; concurrent validity (r = .30 with the Taylor Manifest Anxiety Scale) [14]; and, the capacity to discriminate between clinical and non-clinical samples and anxiety and other psychiatric disorders [14]. Cronbach’s alpha for the SAS in this study was .83.

#### Patient health questionnaire (PHQ)

Participants completed the two-page version of the PHQ, which consists of 9 self-report items covering the DSM-5 diagnostic criteria for Major Depressive Disorder and Other Depressive Disorder and 22 items relating to the criteria for Panic Disorder and Other Anxiety Disorder [3].

To qualify for a Panic Disorder diagnosis, an individual has to first identify as having *“had an anxiety attack, suddenly feeling fear or panic”* within the last 4 weeks. Additionally, they must also endorse that such attacks have happened before, that some of them “come out of the blue” and that these attacks either bother them a lot or that they are worried by the prospect of having more. Finally, they have to endorse four out of eleven somatic symptoms as having been present during their last attack [3].

To qualify for a diagnosis of other anxiety disorders, an individual has first of all to endorse “*feeling nervous, anxious, on edge, or worrying a lot about different things”* on more than half the days over the last four weeks. Additionally, they also have to endorse three of six other anxiety related symptoms (e.g., *“trouble concentrating on things such as reading a book or watching TV”*) as occurring with at least similar frequency.

Spitzer et al. [4] report that the PHQ has 63% sensitivity and 97% specificity when compared with diagnoses made by mental health professionals. The implications of these figures for the current study are discussed in the Data Analysis section below.

### Data analysis

The primary objective in analysis was to examine the impact on sensitivity and specificity of setting the clinical cut-off score for an anxiety diagnosis at different points.

As a precursor to this analysis, however, it was important to reflect on the accuracy of the PHQ diagnoses on which they were based. First, while all members of the clinical sample reported that they were currently receiving treatment for anxiety, this did not necessitate that all would currently satisfy the criteria for an anxiety diagnosis. In an unspecified number of cases, one would expect symptom reductions due to treatment (which may have been either pharmaceutical or psychotherapeutic in nature) to be such that, while treatment might still be continuing, those individuals would no longer meet diagnostic criteria. Additionally, there is the question as to the number of false positives and false negatives that are likely to have occurred in the PHQ diagnoses. Using the sensitivity (63%) and specificity (97%) figures reported by Spitzer et al. [20], it is possible to estimate the approximate number of false positives/negatives that are likely to have occurred in each subsample (Table 2).

**Table 2:** Approximate numbers of false positives and negatives anticipated in PHQ diagnoses by sample

PHD Diagnosis / Original Sample	True Positives	False Positives
Positive Clinical (*n* = 63)	61–62	1–2
Positive Community (*n* = 32)	27	5
	True Negatives	False Negatives
Negative Clinical (*n* = 78)	42–43	35–36
Negative Community (*n* = 178)	161	17

On the basis of these estimates, false PHQ diagnoses can be expected to offer little concern amongst the Positive Clinical subsample. Similarly, false negatives in the Negative Community sample represent no more than 10% of the sample. Amongst the Negative Clinical sample, however, around 45% of the sample are likely to be false negatives, severely compromising the ability of this sample to serve as a test of the SAS’s reliability.

Given,the unreliability of PHQ diagnoses in this subsample, the approach taken in setting the cut-point for the SAS was to combine the sensitivity figures achieved in the Positive Clinical sample with the specificity figures achieved in the Negative Community sample. (This approach, of combining a positive clinical sample with a negative community sample, mirrors that used by Zung [18] when setting the currently recommended cut-off point). This approach is entirely compatible with the Youden Index and ROC curve methods described above, which solely require sensitivity and specificity figures as input. Other methods which involve comparing the overall numbers of correct and incorrect assignations (i.e. true positives and correct rejections versus misses and false positives) were not considered due to difficulty in determining the relative weighting appropriate to clinical and community samples.

All analyses were conducted using IBM SPSS Statistics version 25. The area under the ROC curve was calculated using the non-parametric method [27].

## Results

The number of participants within each sample meeting PHQ criteria for Panic Disorder and for Other Anxiety Disorder is detailed in Table 3. Overall, the proportion of participants satisfying PHQ criteria for some form of anxiety disorder in the clinical and community samples were 44.7 and 15.2% respectively.

**Table 3:** Number of PHQ Anxiety Disorder Diagnoses by Sample

	Clinical Sample (*n* = 141)	Community Sample (*n* = 210)
Panic Disorder Only	14	5
Panic & Other Anxiety Disorder	19	6
Other Anxiety Disorder Only	30	21
Total Anxiety Disorder Diagnoses	63	32

On the basis of these PHQ screenings, the clinical and community samples were each further split into those receiving a positive diagnosis of some sort and those who did not. The mean SAS scores for each of these four subsamples are detailed in Table 4. Within both samples, SAS scores for the positive subsample were significantly higher than those for the negative subsample. Within the clinical sample, this was confirmed by an independent samples *t*-test, *t*(104.4) = 6.14, *p* < .001. For the community sample, severe problems with skewness and kurtosis in the subsample screening negative on the PHQ rendered the *t*-test invalid. However, a Mann Whitney *U*-test confirmed that there was a significant difference between the sub-samples SAS scores, *U* = 673.5, *p* < .001.

**Table 4:** Mean SAS scores for participants screening positive and negative for anxiety disorders on the PHQ

SAS Mean Score (*SD*)	Clinical Sample	Community Sample
PHQ Positive Anxiety Diagnosis	44.93 (9.66)	44.28 (9.07)
No PHQ Diagnosis	36.21 (6.50)	32.20 (6.39)

As detailed in the Data Analysis section, subsequent analysis focussed solely on the Positive Clinical and Negative Community subsamples. Sensitivity and specificity figures within these subsamples (detailing the extent to which SAS diagnoses were in agreement with those of the PHQ) for progressive cut-off points varying from 34 to 42 are detailed in Tables 5 and 6 respectively.

**Table 5:** Sensitivity: Percentage of cases with positive anxiety diagnoses on the PHQ also diagnosed on the SAS by cut-off point

Cut-off point	Clinical Sample (*n* = 63)	Community Sample (*n =* 32)
34	93.7	93.7
35	90.5	90.6
36	88.9	84.4
37	84.1	75.0
38	81.0	72.9
39	77.8	68.7
40	74.6	62.5
41	66.7	59.4
42	60.3	59.4

**Table 6:** Specificity: Percentage of cases with no diagnosis on the PHQ that similarly would receive no diagnosis on the SAS analysed by cut-off point

Cut-off point	Clinical Sample (*n* = 78)	Community Sample (*n =* 178)
34	37.2	58.4
35	41.0	66.3
36	46.2	74.7
37	53.8	80.9
38	56.4	84.8
39	59.0	88.2
40	69.2	90.4
41	76.9	92.1
42	78.2	94.4

**Fig. 1:**
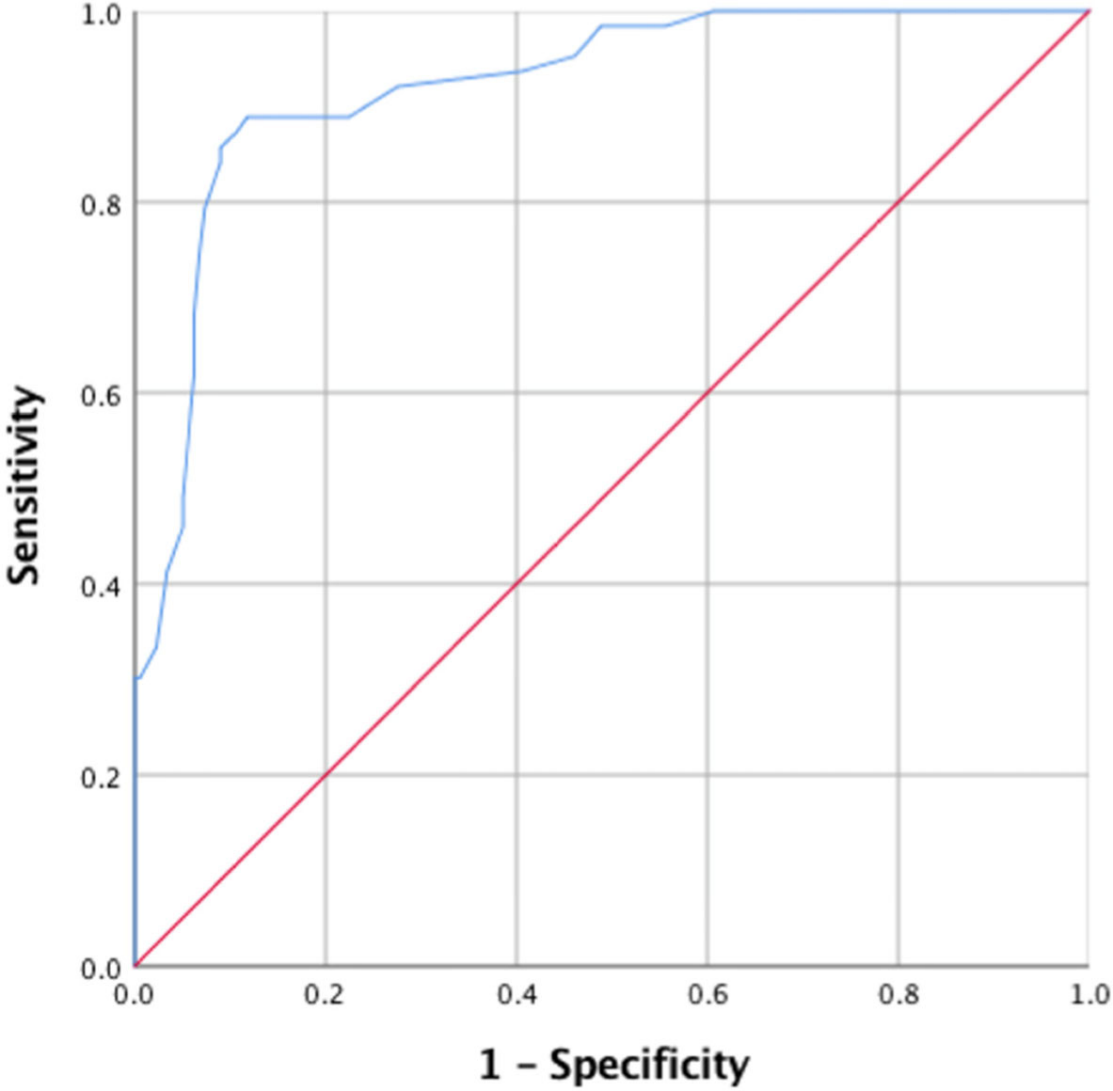
ROC curve for the combined Positive Clinical and Negative Community samples (blue line). Sensitivity of the SAS in the Positive Clinical subsample is graphed against 1 - the specificity in the Negative Community subsample for each potential SAS cut-off point

The ROC curve that results using these two samples is shown in Fig. 1. The area under the curve equals .89 (95% Confidence Interval: .84–.94).

Utilising these two samples, Table 7 details what each of the mathematical methods reviewed (the Youden Index and the ROC curve) would suggest regarding the optimum cut-off point for the SAS, together with the associated sensitivity and specificity figures. In both cases, the optimum mathematical cut-off sits between the current and the original scale cut-offs: 39 using the Youden Index and 38 using the ROC curve. Table 8 compares the optimum Youden Index and ROC curve values obtained with those of the current cut-off of 36 and the original cut-off of 40. While the Youden Index method favours the original cut-off, the current cut-off slightly favours the current point.

**Table 7:** SAS Cut-off figures recommended by mathematical methods, together with the resulting sensitivity and specificity scores

Mathematical Model	Optimum Cut-Off	Sensitivity	Specificity
Youden Index	39	78%	88%
ROC Curve^a^	38	81%	85%

^a^ In this instance both ROC curve methods, selecting the value for which the curve comes closest to the point (0,1) and selecting the value closest to the point where the curve intersects the line Se = Sp, yield the same result

**Table 8:** Comparison of mathematical values obtained with optimum cut-off versus the current and original cut-offs recommended by Zung*

Mathematical Model	Optimum(39/38)	Current (36)	Original (40)
Youden Index	.660	.636	.650
ROC Curve: distance from point (0,1)	.243	.271	.276

* For the Youden Index higher values are desirable, for the ROC curve lower values

## Discussion

With the existing cut-off score of 36, the SAS achieved a sensitivity of 89% in the positive clinical sample, a figure identical to that recorded by Zung in the research on which this cut-off was based [18]. However, specificity in the community sample was only 75%, indicating that with the existing cut-off, there is a one in four chance of a false positive. (Zung’s research did not measure specificity as no formal diagnoses were undertaken amongst his normal adult sample [18]).

Mathematical methods such as the Youden Index and ROC curve methods suggest a higher cut-off might be appropriate, with 38 emerging as the leading candidate. From a purely mathematical perspective, there is little to choose between the original cut-off of 40 and Zung’s current recommendation [18]. However, these mathematical models do not discriminate between false negatives and false positives, nor do they make any allowance for the prevalence of the disorder under consideration, factors which, along with the purpose of testing, are crucial if the value of the test under consideration is to be maximised [28]. A reported 11.8% of the Australian adult population suffer from some sort of anxiety disorder during the course of any one year [29]. On this basis, applying the sensitivity and specifity figures obtained from the positive clinical and negative community sample, then for a representative adult sample of 100, the expected number of false positives and false negatives at the different cut-offs is as detailed in Table 9.

**Table 9:** Expected numbers of false positives and false negatives per 100 cases using the original and current SAS cut-offs

Cut-off	False Negatives	False Positives	Total Misdiagnoses
Original: 40	3^a^	9	12
Current: 36	1	22	23

^a^Note if the 62.5% sensitivity recorded in the positive community sample were applied here this figure would increase to 4–5 cases

Examining these results reveals that the case for increasing the recommended clinical cut-off score is far less evident for the SAS than the SDS; see [20] for comparative figures]. While the current cut-off is forecast to produce a sizeable number of false negatives, this has to be balanced against the risk of a number of clinical cases going undiagnosed if the cut-off is raised. It should also be noted that amongst the positive community sample, sensitivity falls away more rapidly as the cut-off increases and is only 63% for the original cut-off of 40. While the results for this sample are somewhat compromised by the low sensitivity of the PHQ, nevertheless the importance of identifying potential sufferers from anxiety who have not yet been diagnosed argues for caution in not setting the cut-off point at too high a level.

### Limitations

A significant limitation of this study is the fact that the diagnoses on which the SAS sensitivity and specificity figures are based are made on the basis of self-report (namely the criterion-referenced PHQ) rather than clinician conducted interviews. While reported sensitivity and specificity figures for the PHQ itself suggest that errors in diagnosis for the two subsamples used in analysis would be few, the fact that the results for the Positive Community and Negative Clinical samples had to be excluded, limits the degree of confidence that can be placed on these results.

A further potential issue is that similarities between the two self-report measures may inflate the correlations between diagnoses. However, the fact that the PHQ is a criterion-referenced rather than norm-referenced lessens this concern.

Finally, it should be noted that the perspective taken by this study is broad-based: no distinction is made between different types of anxiety disorders, nor indeed between demographic sub-groups (e.g. differences in gender or by age-group). While this is a common approach in setting cut-off scores, it does leave the generalisability of sensitivity and specifity figures open to question.

## Conclusions

Ultimately, which SAS cut-off is most appropriate depends on the differential costs attached to false positives and false negatives. In a clinical screening situation where false negatives may result in patients not receiving appropriate treatment, there is an argument for retaining the current cut-off recommended by Zung [18]. However, the larger number of overall misdiagnoses that result form a strong argument for reverting to the original cut-off of 40 when using the SAS in a research context.

Finally, it should be noted that this study again demonstrates the value of the SAS as a screener for anxiety disorders. Not only is the area under the SAS ROC curve indicative of a discriminating test, but sensitivity and specificity figures more than bear comparison with those reported for the DASS anxiety index (e.g., [16, 30, 31]). This is important in that, while there are many scales available to screen for anxiety disorders, the SAS appears to be one of the more successful scales at tapping into the specific nature of anxiety symptoms, rather than the more general negativity of emotions common to both depression and anxiety [7–11, 14, 16]. In conjunction with the scale’s continued widespread research use, the need to settle on appropriate cut-off scores is paramount.

## Data Availability

The study data is available from the corresponding author on application.

## Abbreviations

DASS: Depression Anxiety Stress Scale
DSM: Diagnostic and Statistical Manual of Mental Disorders
PHQ: Patient Health Questionnaire
ROC: Receiver Operating Characteristics
SAS: Zung Self Rating Anxiety Scale
